# Surface-mediated spontaneous emulsification of the acylated peptide, semaglutide

**DOI:** 10.1073/pnas.2305770121

**Published:** 2024-01-16

**Authors:** Qi Li, Vasudev Tangry, David P. Allen, Kevin D. Seibert, Ken K. Qian, Norman J. Wagner

**Affiliations:** ^a^Department of Chemical and Biomolecular Engineering, Center for Neutron Science, University of Delaware, Newark, DE 19716; ^b^Eli Lilly and Company, Indianapolis, IN 46225

**Keywords:** spontaneous emulsification, acylated peptides, semaglutide, kinetics, stability

## Abstract

Acylated peptides composed of glucagon-like peptide-1 receptor agonists modified with a fatty acid side chain are an important class of therapeutics for type 2 diabetes and obesity but are susceptible to an unusual physical instability in the presence of hydrophobic surfaces, i.e., spontaneous emulsification, also known as ouzo formation in practice. In this work, light scattering, small-angle X-ray scattering, and circular dichroism measurements are used to characterize the physical properties of the semaglutide colloidal phase, including size distribution, shape, secondary structure, internal structure, and internal composition, as a function of solution physico-chemical conditions. The existence and size of the colloids formed are successfully predicted by a classical Rayleigh model, which identifies the parameters controlling their size and formation. Colloid formation is found to be catalyzed by hydrophobic surfaces, and formation rates are modeled as an autocatalytic reaction, enabling the formation of a master curve for various surfaces that elucidates the mechanism. Surfaces differ due to differences in surface wettability, which can be correlated with Hansen solubility parameters. This work provides insights into this unusual colloidal phenomenon and guides the peptide synthesis process and drug product formulation in the pharmaceutical industry.

## Significance

We identify the fundamental mechanisms of an undesired instability known as “ouzo” formation in a class of therapeutics used for treating type 2 diabetes and obesity. Spontaneous emulsification in solutions of acylated peptides in the presence of hydrophobic surfaces is elucidated as a function of physico-chemical conditions and surface hydrophobicity as characterized by Hansen solubility parameters. Quantitative prediction of the colloidal size is demonstrated using the classical Rayleigh theory, while formation rates reduce to a master curve dependent on the surface hydrophobicity and stirring rate. We demonstrate that colloidal physics and molecular thermodynamics provide quantitative predictions of the colloidal droplet size and qualitatively rank formation rates, thereby improving our understanding of this important class of therapeutic molecules.

The global epidemic rise in type 2 diabetes and obesity has been well recognized. As economic development and urbanization lead to lifestyle changes such as reduced physical activity and increased food intake, the number of patients worldwide has increased significantly over the last two decades. Substantial medical need and economic burden are among the most obvious societal consequences. Therefore, the identification of novel, safe, and effective glucose- and weight-lowering therapeutic agents is of the utmost importance. Currently, the Food and Drug Administration and European Medicines Agency have approved several treatments against type 2 diabetes and/or obesity, with examples including semaglutide (SMG) (1), liraglutide and tirzepatide (1–3). These molecules are peptide mimetics derived from native gastrointestinal hormones (4). Furthermore, these peptides are modified by conjugation to fatty acid chains, promoting reversible binding to albumin, thereby increasing the peptide biological half-life (5, 6).

The therapeutic value merits study of their physical stability in their manufacturing, formulation, and delivery. Aggregation of peptides is one of the most common degradation pathways observed in almost all phases of drug product development. Aggregation can take several different forms, and the term is often used to describe a variety of processes during which peptide molecules assemble into larger species. The aggregates can be amorphous or structured such as amyloid fibrils. In severe cases, it leads to a loss in activity and/or induces toxicity and immunogenicity. Many examples of aggregation were initiated by a loss of secondary structure (unfolding). For example, SMG and liraglutide form fibrils consisting of β-sheets with aging (7) or with small changes in the manufacturing process (8). In recent years, extensive research has been undertaken to increase our understanding of the mechanisms by which peptide aggregation occurs.

Another problematic solution instability that is distinct from the aforementioned aggregation in peptides is spontaneous emulsification into high molar mass colloidal structures in formulation when in physical contact with specific container surfaces, often termed “ouzo formation” in practice. Such colloid formation adversely affects manufacturing and delivery. The work presented here reports our investigations on the nanoscale solution microstructure and colloidal formation rates as a type of surface-mediated spontaneous emulsification (*SI Appendix*, *Glossary of Definitions*), using a model peptide, SMG. SMG is a commercialized therapeutic peptide used for diabetic and obesity treatment. Structurally, it is a glucagon-like peptide-1 mimetic, conjugated with a stearic diacid through acylation. Different from the self-association process that is known to form oligomers in stable solution, spontaneous emulsification (9) is a microphase separation that affects drug product safety, efficacy, and quality. We found that this instability has conceptual similarity to the ouzo effect in colloid and interface science, but unlike the classical case where the emulsification is driven by external fluctuations in solubility (10), here it is catalyzed by surfaces with specific properties. The results of these investigations provide valuable understanding to guide peptide synthesis, formulation, manufacturing process, and storage.

## Results and Discussion

### Surface-Mediated Instability.

**Fig. 1.**
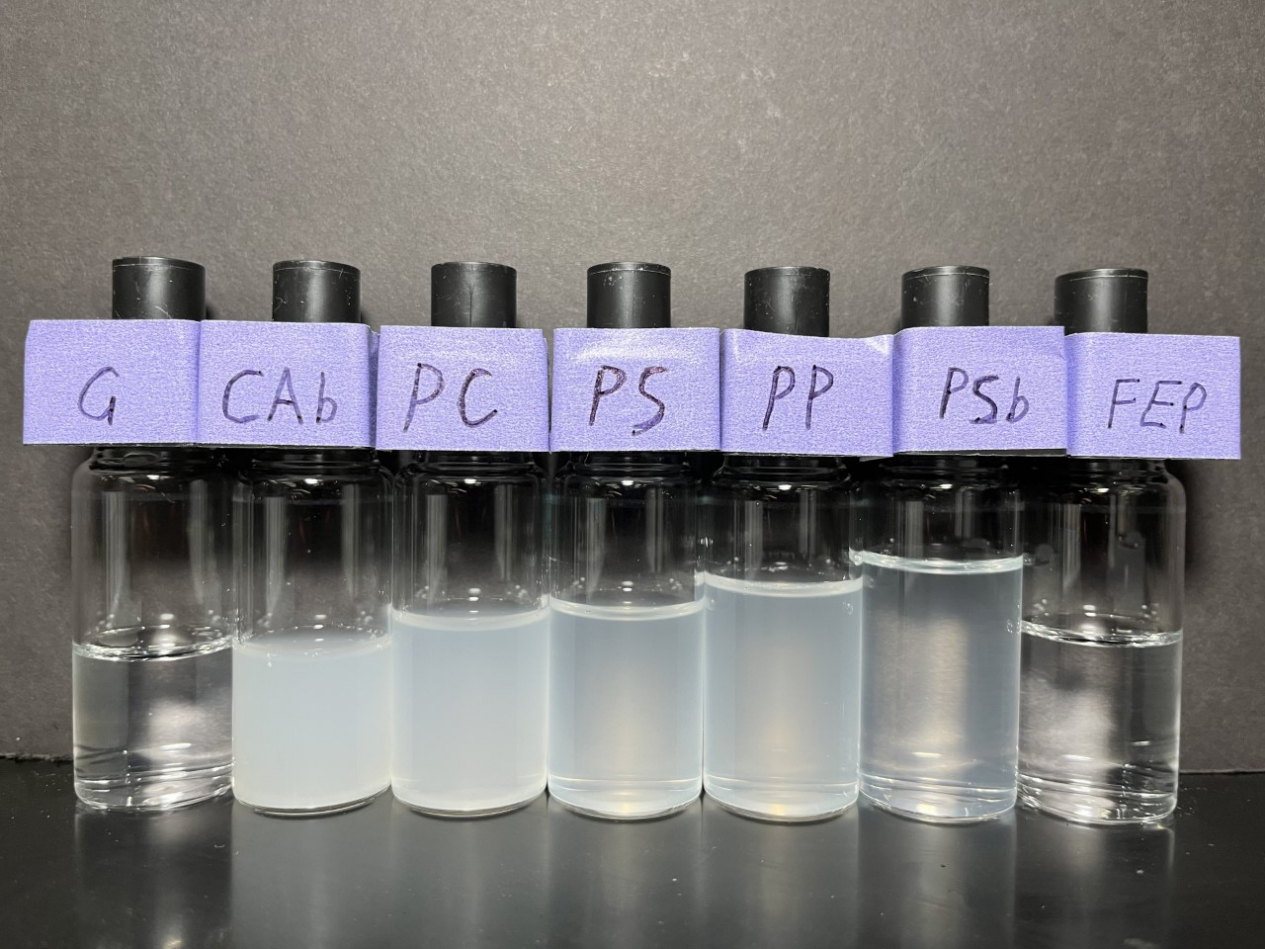
Images of solutions (transferred to glass vials for measurements) studied, some showing surface-induced spontaneous emulsification (“ouzo” effect) of SMG in the presence of different materials. Samples were collected from the SMG stirring test (250 rpm at 25 °C, with PTFE stirring bar) or static test with a fixed surface-to-solution volume ratio (StV = 1.0 cm^−1^) for different amount of time. From *Left* to *Right*: (G) stirring in a glass vial for 4 wk; (CAb) stirring with CAb for 6 h; (PC) stirring in a PC container for 8 h; (PS) stirring in a PS container for 8 h; (PP) stirring in a polypropylene container for 8 h; (PSb) quiescent with PSb for 3 wk; (FEP) stirring in a FEP container for 6 wk.

The overall, qualitative results of the stirring and static tests can be summarized from visual observations reported in Fig. 1. Solutions became visibly cloudy with time in polystyrene (PS), industrial polypropylene (PP, which importantly is known to contain additives as described later), and polycarbonate (PC) containers as well as for solutions in glass (G) containers with added PS beads (PSb) or cellulose acetate beads (CAb). In contrast, for fluorinated ethylene polypropylene (FEP) and G containers, static or stirred (even for weeks), the peptide solutions remained clear, indicating that the observed physical instability is sensitive to and “catalyzed” by specific hydrophobic surfaces. Further, these observations demonstrate that the instability is insensitive to the presence of the air-water interface, which is distinct from prior observations reported for insulin solutions. For example, Langer et al. (11) discovered the aggregation of bovine Zn-insulin upon agitation that was triggered by either a Teflon surface or the air-water interface. Importantly, in our experiments, the time required for the onset of turbidity differs with surface materials and the rate of mechanical agitation. For samples in a PS container, cloudiness was observed in 30 min with stirring (see *SI Appendix*, Fig. S1 for images) while under static conditions, visible cloudiness was not observed until ~2 wk. For PP, the SMG solution remains clear until around 20 h with stirring, while at rest, cloudiness was not observed until 2 mo. The differences between surface materials and stirring rates indicate that the instability is mediated by peptide-surface interactions.

**Fig. 2.**
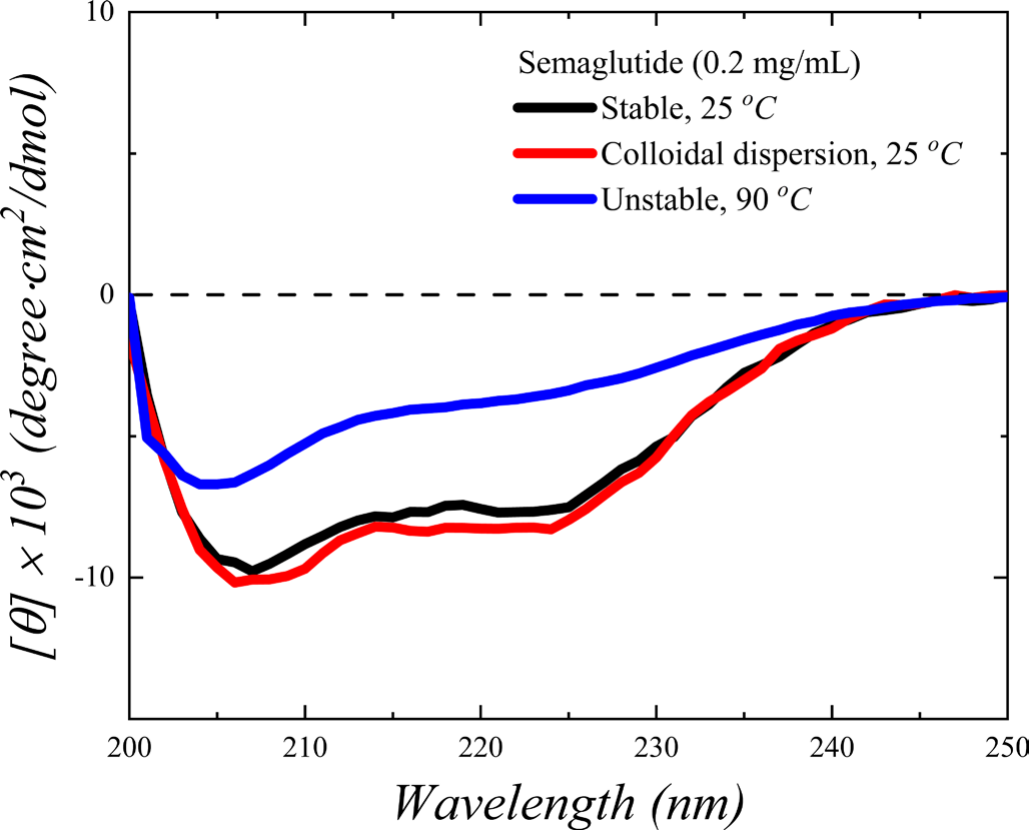
CD spectra of the SMG clear (black) and colloidal dispersion (red, stirring test of 20 mg/mL SMG in PS for 16 h) solutions after X100 dilution to 0.2 mg/mL at 25 °C. Note that the colloidal droplets are stable with dilution, verified by DLS. The spectra of a same 0.2 mg/mL SMG exposed to 90 *°*C for 2 min (blue) serve as a reference for a partially denatured sample.

Circular dichroism (CD) showed no change in the secondary structure of peptide between the clear and cloudy solutions (Fig. 2) in comparison with a sample exposed to high temperature, where the amount of α-helix structure significantly decreases. The α-helix structure and its extent remain constant without β-sheet formation during spontaneous emulsification, confirming that significant denaturation, such as observed at high temperature, is not occurring. Such behavior distinguishes the observed instability from previously reported aggregations, such as fibrillation [SMG (8), liraglutide (12), and insulin (13, 14)] and spherulite [insulin (15)], which were accompanied by structure transitions from α-helices to β-sheets and interpreted as a signature of partial denaturation. The retention of the protein’s secondary structure is also confirmed by a Kratky plot of results from small-angle X-ray scattering (SAXS) measurements (*SI Appendix*, Fig. S2), where the comparatively large size of the emulsion droplets means that they don’t contribute to the scattering features from the oligomers remaining in solution. The colloidal dispersion formed is stable over time (measured for 10 mo) and cannot be removed by centrifugation (20,000 g) or dilution (detectable up to 100 times), or temperature variations (5 to 40 °C). Further, light-scattering size measurements confirmed that the hydrodynamic size of the colloids does not change when subjected to these treatments, providing evidence that once formed, the colloids are remarkably stable, as is also the case for ouzo dispersions.

**Fig. 3.**
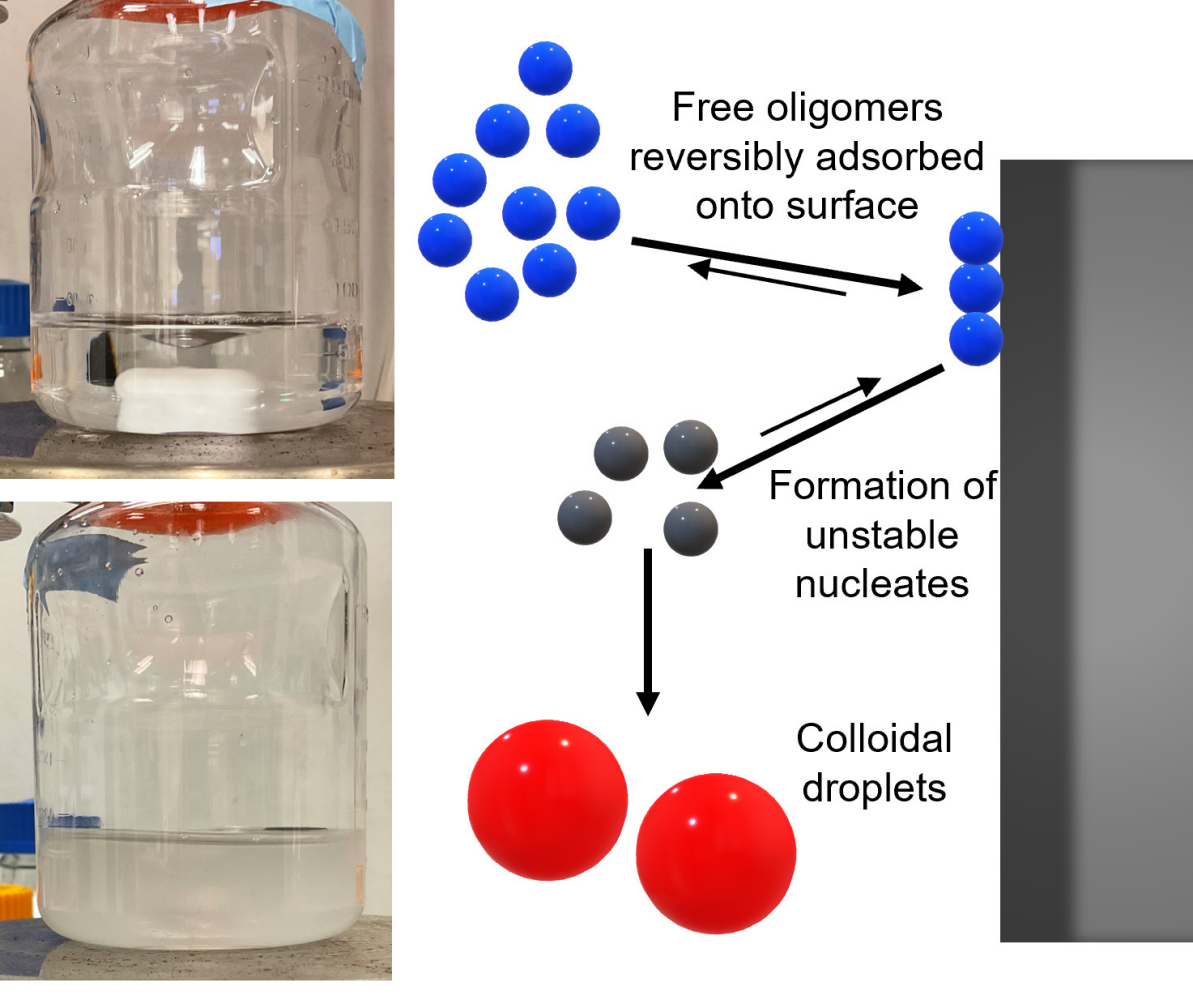
Schematic of the proposed mechanism for the surface-mediated spontaneous emulsification process (ouzo) effect. Photos of the SMG stirring experiment are shown, including one taken at the beginning of stirring (*Upper* *Left*) and one taken after stirring for 8 h (*Lower* *Left*) in a PS container.

As the spontaneous emulsification observed here is not accompanied by any secondary structure change and occurs in the absence of surfactant, we remark that this instability is conceptually similar to the “ouzo effect,” which is named by Vitale and Katz after a popular Greek beverage (10). In a typical ouzo effect, the spontaneous emulsification is induced by external fluctuations such as introducing a solvent with a different solubility to the solute. Fig. 3 is a schematic of a proposed mechanism of the surface-mediated ouzo effect, involving surface adsorption/desorption of stable self-associated oligomers, surface desorption of an unstable nucleate, and colloidal droplet growth in solution. The first two steps concerning adsorption and desorption involve peptide-surface interaction and possibly surface-mediated coacervation, which leads to production of unstable nucleates in solution. These nucleates spontaneously grow to form highly stable colloids rich in SMG and the solution becomes turbid. As noted, the colloidal dispersion formed is stable, unlike coacervates or emulsions, and is turbid, unlike microemulsions, and strongly resembles the classic ouzo. Further evidence for the mechanism of this surface-mediated, spontaneous emulsification is provided in the following sections.

### Light Scattering and SAXS Characterizations.

**Fig. 4.**
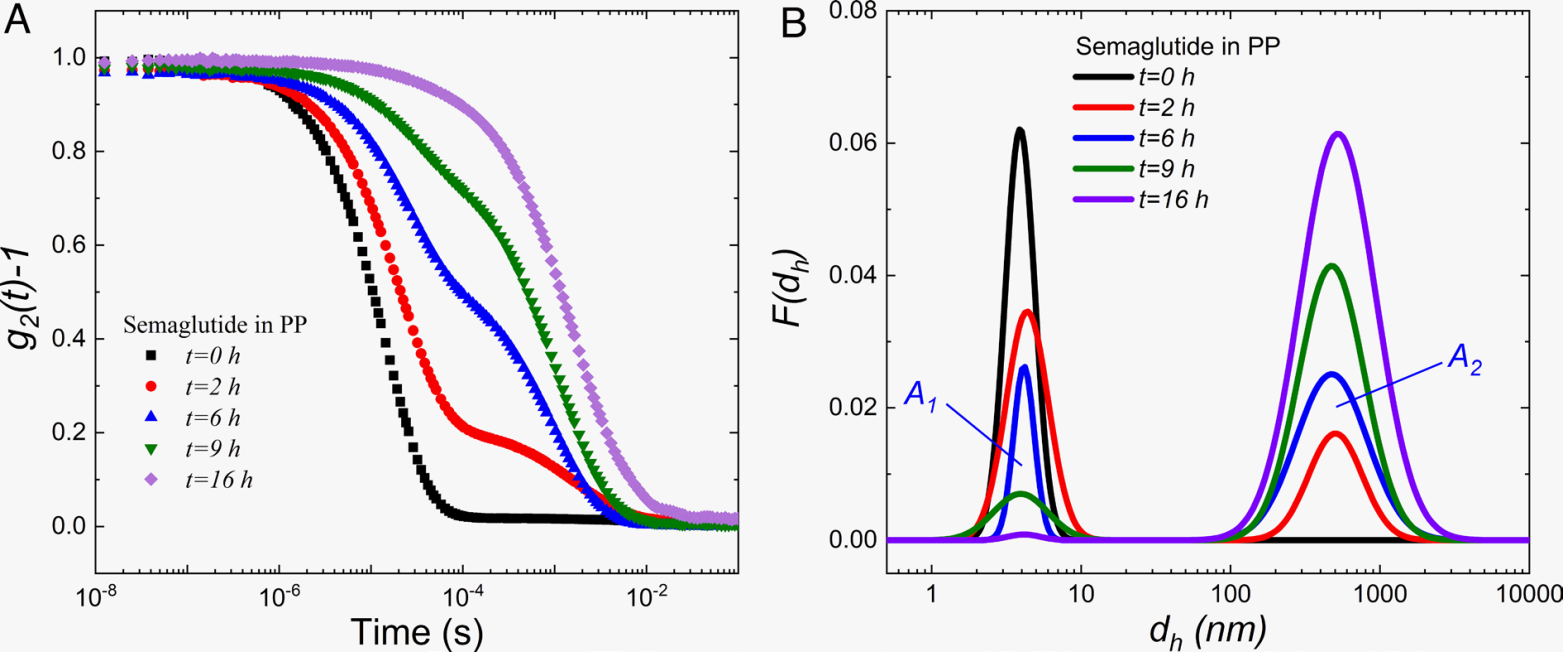
Representative results for (*A*) intensity autocorrelation function, $g_{2}\left(t\right)-1$ , as a function of delay time for the SMG stirring test in a polypropylene container (PP); samples taken at the indicated times during stirring; and (*B*) intensity-averaged size distributions during the stirring test.

**Fig. 5.**
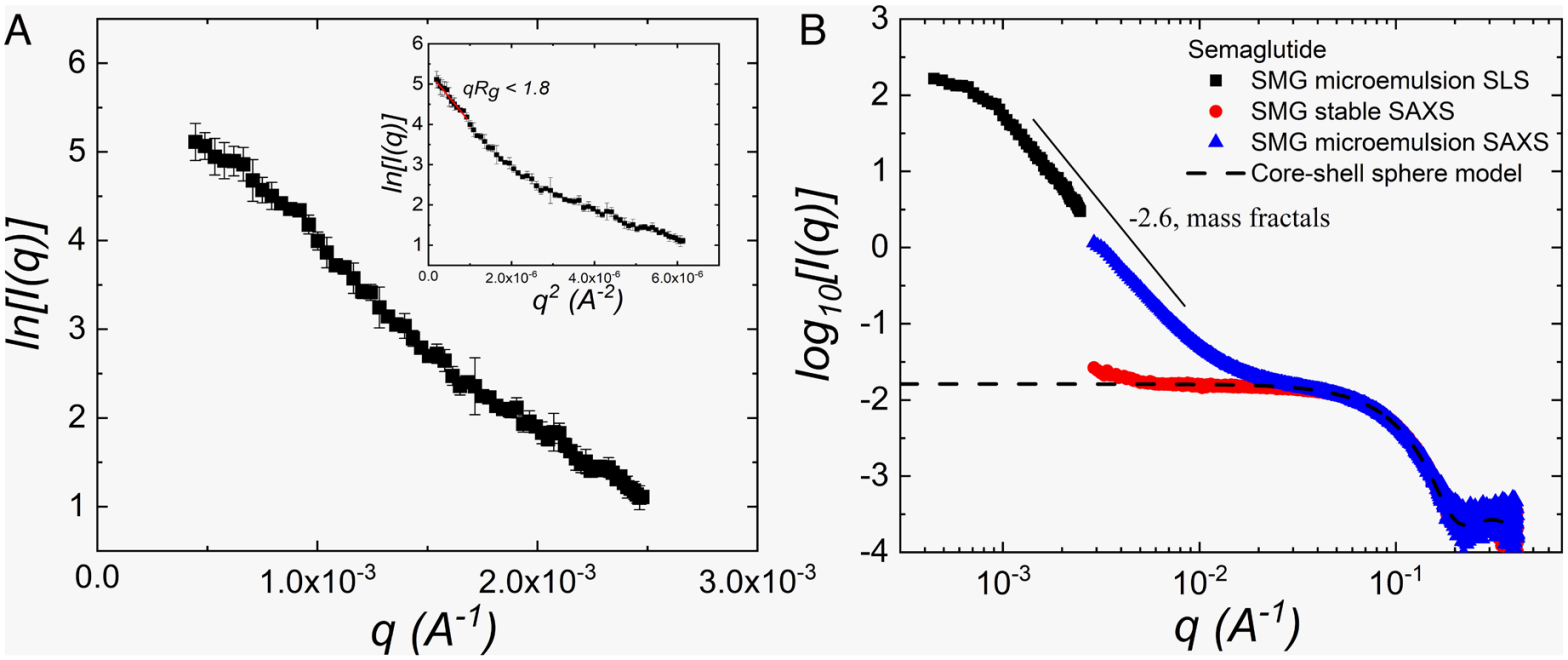
(*A*) SLS results on SMG microemulsion (stirring test of 20 mg/mL SMG in PS for 16 h at 25 °C) after dilution from 20 to 2 mg/mL. Scattering angles range from 10° to 150°. The *Inset* shows the Guinier plot analysis ( $\text{ln}\left[I\left(q\right)\right]\;\text{vs}.\;q^{2}$ , Eq. **8**) from the SLS data. The Guinier region was chosen to be when $qR_{g}<1.8$ , following the criteria for folded protein. (*B*) SLS-SAXS combined results, ${\text{log}}_{10}\left[I\left(q\right)\right]\;\text{vs}.\;q$ , for SMG microemulsion (2 mg/mL) and SMG stable solution (only SAXS, 2 mg/mL). The solid guideline is for showing the Porod slope of −2.6, indicating a mass fractals internal structure. The gray dashed line is a polydisperse sphere model fit of the SAXS data with the fit details presented in *SI Appendix*.

The size and size distribution of stable samples were measured by dynamic light scattering (DLS), while the 3D cross-correlation technique implemented by LS Spectrometer™ was used for turbid samples. DLS provides raw data in the form of the intensity autocorrelation function (ACF), $g_{2}\left(t\right)$   , vs. delay time *t*; an example is shown in Fig. 4*A* for a sample stirred in a polypropylene container (PP). With stirring, long-time relaxation modes appear in the ACF as a second decay in $g_{2}\left(t\right)$   in the long-time limit. The hydrodynamic diameter $d_{H}$   and its distribution are calculated via the CONTIN method (16, 17) and shown in Fig. 4*B*, assuming the Stokes–Einstein–Sutherland equation (see *SI Appendix* for more information) (18, 19). Results for the initial, clear solution are shown in Fig. 4*B* (“*t* = 0 h” curve), where a mono-modal distribution is observed with $d_{H}=4.1\pm 0.4\;\text{nm}$ . Analysis of a Zimm-plot (*SI Appendix*, Fig. S3) confirms that this hydrodynamic diameter corresponds to self-association of the acylated peptide into oligomers ranging from tetramers to hexamers. SAXS characterization of these complexes (Fig. 5*B*) yield the radius of gyration to be $R_{g}=1.9\pm 0.1\;\text{nm}$ . As a result, the ratio of ${2R}_{g}/d_{H}∼0.91\pm 0.09$ indicates that the shape of the oligomer in solution is between constant-density sphere ( ${2R}_{g}/d_{H}=\sqrt{3/5}\;\sim \;0.77$ ) and hollow sphere ( ${2R}_{g}/d_{H}=1.00$ ). As a volume-scattering method, the overlapping of the SAXS spectra at high-q ( $>0.05\;{Å}^{-1}$ ) indicates a common structure for length scales of around 6 nm and below for both the clear oligomer and turbid colloidal solutions, which suggests that the colloidal droplets consist of aggregated oligomers. Furthermore, a polydisperse sphere model was fit the SAXS spectra obtained from the SMG oligomer solution to reveal the structure of the oligomer molecule (details in *SI Appendix*). The fitted radius ( 2.1±0.3 nm ) agrees with DLS and SLS (Static light-scattering) measurements.

Analysis of the DLS measurements, presented in Fig. 4*B*, shows that spontaneous emulsification due to stirring in the PP container leads to the formation of micron-sized colloidal droplets whose number increases with stirring time, commensurate with a loss of oligomers in solution. Importantly, a well-defined bimodal distribution is observed with characteristic, intensity-averaged sizes of 4.1±0.4 nm and 514.5±26.8 nm , indicating that the droplets formed are colloidally stable. The lack of intermediate sizes suggests that either the micron-sized colloidal droplets directly originate from the surface, or that they form rapidly in solution from nucleation sites desorbed from the surface; further elucidation of this mechanistic step is the subject of ongoing research.

The spontaneous emulsification we observe resembles aspects of a prior report of fibrillation in insulin solutions (11), also in the presence of hydrophobic surfaces. A two-step mechanism was proposed where the rate-limiting step is “nucleus” formation and desorption from the interface followed by very rapid growth in solution. We propose a similar mechanism; however, differences in the structure of material formed in the colloidal phase (e.g., fibrils vs. stable colloidal dispersions) and the details of the kinetics are due to differences in the molecular species involved. In our work, using the bimodal size distribution determined from DLS, the relative amount (scattering intensity-averaged) of colloidal droplets to oligomeric molecules provides a metric for tracking the rate of ouzo formation. As indicated in Fig. 4*B*, the areas under the two peaks are defined as $A_{1}$   and $A_{2}$   , corresponding to the intensity-averaged amount of oligomeric and colloidal species. It is noteworthy that the large colloidal droplets scatter light much more strongly than the stable oligomers so the ratio is not directly proportional to the mass in each state. For example, simple Rayleigh–Gans–Debye scattering theory (20, 21) suggests that a 514.5 nm particle has around $10^{12}$ times more scattering intensity than a 4.1 nm oligomer, although this is an overestimate as the colloids are larger in size than the range of validity of the theory and their optical scattering length density may be lower than the oligomer as the droplets contain a significant amount of buffer. Nonetheless, the intensity-averaged size distribution is much more sensitive to the presence of the colloidal phase as compared to a number-averaged size distribution, which would be determined by direct particle imaging, for example.

A bimodal size distribution is observed for all turbid samples and importantly, the two characteristic sizes are found to be independent of surface materials or rate of stirring. Further investigations on buffer conditions, however, reveal that the size of the colloidal droplets depends on salt concentration. The additional sodium chloride in buffer (“high salt”: 373 mM NaCl and 8 mM ${\text{Na}}_{2}{\text{HPO}}_{4}\cdot 2H_{2}O$ ) decreases the size of the colloidal droplets to 386.6±63.5 nm , while diluting the salt concentration (“low salt”: 70 mM NaCl and 4 mM ${\text{Na}}_{2}{\text{HPO}}_{4}\cdot 2H_{2}O$ ) increases the size to 1,136.8±77.5 nm . On the other hand, NaCl concentration only weakly affects oligomerization, where at the high-salt condition, the molecular complexation of SMG has a slightly increased $d_{H}=4.9\pm 0.2\;\text{nm}$ , while at the low-salt condition, it is slightly decreased $d_{H}=4.0\pm 0.3\;\text{nm}$ . The trend of self-association with salt agrees with the current understanding where the addition of salt promotes the formation of multimers (22). Meanwhile, the opposite trend observed for the size variation of the colloidal droplets with salt condition follows the predictions of the Rayleigh charge-limiting size model (23), as will be discussed in detail in the following section.

The shape and structure of the colloidal droplets can be ascertained by comparison of the hydrodynamic size ( $d_{H}$   ), with the radius of gyration ( $R_{g}$   ) as ${2R}_{g}/d_{H}$   . A plot of the scattering intensity, I   , as a function of scattering vector, q   , measured by SLS on the SMG colloidal formed in a PS container with stirring is shown in Fig. 5*A*. The slope of the Guinier fitting (Eq. **1** and Fig. 5 *A*, *Inset*) yields $R_{g}=195.5\pm 2.4\;\text{nm}$ , such that ${2R}_{g}/d_{H}∼0.76\pm 0.05$ , indicating that the shape of the aggregates is consistent with a constant density sphere ( ${2R}_{g}/d_{H}=\sqrt{3/5}\;∼\;0.77$ ) (24).

Due to the difference in wavelength, light scattering focuses on the length scale of a few hundred nanometers, while SAXS can reach smaller length scales. The combination of light scattering and SAXS can therefore provide information on the internal structure of the colloidal droplets. Fig. 5*B* presents the measurements as I(q) vs. q . The observed slope of 2.6 in the log-log plot at lower scattering vectors is interpreted as a mass fractal dimension (25) and is typical of a dense, gel-like network internal to the SMG colloidal droplets. Further analysis of the internal composition is determined from the analysis of a Zimm plot from SLS (*SI Appendix*, Fig. S4). In the Guinier region, the molecular weight inside the droplet is estimated to be $\left(4.3\pm 1.8\right)\times 10^{6}\;\text{kDa}$ . Using a density for the SMG of ( $\rho_{SMG}=1.16\pm 0.04\;g/{\text{cm}}^{3}$ ) yields approximately 14.8±5.1 vol % SMG inside the droplet with the remainder being buffer. This result is consistent with the near density match and low relative refractive index of the colloidal droplets as they are primarily composed of buffer.

### Rayleigh Charge-Limiting Size.

For colloidal microemulsions, interfacial tension (IFT) is one of the controlling parameters for the stability of the droplet interface, and IFT drives the system toward macrophase separation. However, acylated peptides such as SMG are inherently charged in buffer due to the high dielectric constant of water and the ionizable groups on the amino acids in a specific pH range. The added salt in the buffer provides additional ions to screen electrostatic interactions in solution. This electrostatic force acts to stabilizing the SMG colloidal droplets against macrophase separation. The important role of electrostatic repulsion is evident from the Zimm plot (*SI Appendix*, Fig. S4) where the second virial coefficient ( $A_{2}\approx 1.7\times 10^{-18}\;m^{3}\approx 6A_{2,\;\text{hard}\;\text{sphere}}$ ) indicates that there is strong repulsive interaction between droplets. From light-scattering experiments, it was found that the droplet size depends on the salt condition, but not on surface materials or the type of the experiment (stirring or static). These experimental observations simplify the parameters controlling droplet size and stability to a balance between IFT and electrostatic interactions.

In 1882, Lord Rayleigh proposed that the limiting size of charged droplets is given by balancing the surface energy with the electrostatic energy:

[1] $$Q=16\pi {\left(2\varepsilon_{0}\gamma d^{3}\right)}^{1/2}.$$

In this result, Q is the charge of the droplets, $\varepsilon_{0}=8.85\times$ $10^{-12\;}F\;m^{-1}$ is the vacuum permittivity, γ is IFT, and d denotes the droplet diameter. The SMG colloidal droplets are in buffer solution, and Gauss’s law (26) enables representing the entire charge in the droplet to be on the droplet surface. Therefore, the Rayleigh charge-limiting droplet size can be rewritten in terms of surface charge density ( σ):

[2] $$d_{\text{Rayleigh}}=\frac{8\varepsilon_{0}\gamma }{\sigma^{2}},$$

where σ can be calculated via the Grahame equation (*SI Appendix*) from the zeta potential, which is measured independently. Due to the peptide and fatty acid chain structure, SMG molecules in their stable form can be modeled as polymer chains. Thus, the IFT, γ , is related to the local compositions and molecular interactions via the Flory–Huggins interaction parameter, χ (27–29).

[3] $$\gamma_{\mathrm{FH}}=\frac{\mathrm{kT}}{b^{2}}{\left(\frac{\chi }{6}\right)}^{1/2}\approx 5.2\;\text{dyn}/\text{cm},$$

[4] $$\text{with}\;\;\chi \approx \frac{v_{\text{SMG}}}{\text{RT}}{\left(\delta_{\text{SMG}}-\delta_{\text{buffer}}\right)}^{2},$$

where $v_{\text{SMG}}$   is molar volume of the molecule and δ   is solubility parameter. Both were calculated from the chemical structure using a group contribution method (30, 31). In the calculation, R is gas constant and k is the Boltzmann constant. T is temperature in Kelvin, and b≈3.7 Å is the Kuhn length, which in the current case was taken to be the average size of amino acids. The model parameter estimations simplify the peptide–peptide interactions and model the chain configuration variations via a lattice model, which applies for the SMG in the semi-dilute concentration regime. The predictions of the Rayleigh model for the charge-limiting droplet sizes are in remarkable agreement with the experimentally measured $d_{H}$ from light scattering (Table 1). We note that the zeta potential is measured as it is difficult to calculate theoretically because of ion condensation and dissociation variations with different buffer conditions. This quantitative agreement between model and experimental results demonstrates that the competition between IFT and surface charge interactions controls the size of the colloidal droplets, and further, can be adjusted by variation in solution electrolyte conditions, as indicated in Table 1.

**Table 1.** Rayleigh charge-limiting droplet sizes and relevant parameters for 20 mg/mL SMG in three different salt/excipient conditions

	Salt condition^*^		
Parameters	141 and 8 mM	374 and 8 mM	70 and 4 mM
Zeta potential (mV)	-23.8±0.5	-19.1±0.2	-25.7±0.3
σ (mC/m^2^)	26.7±0.5	33.0±0.3	17.3±0.2
Prediction: $d_{\text{Rayleigh}}$(nm)	519±27	321±11	1226±31
Experimental: $d_{H}$(nm)	537.3±30.1	386.6±63.5	1,136.8±77.5

^*^Denoting the ratio between sodium chloride and sodium phosphate, respectively.

### Emulsification Rates.

**Fig. 6.**
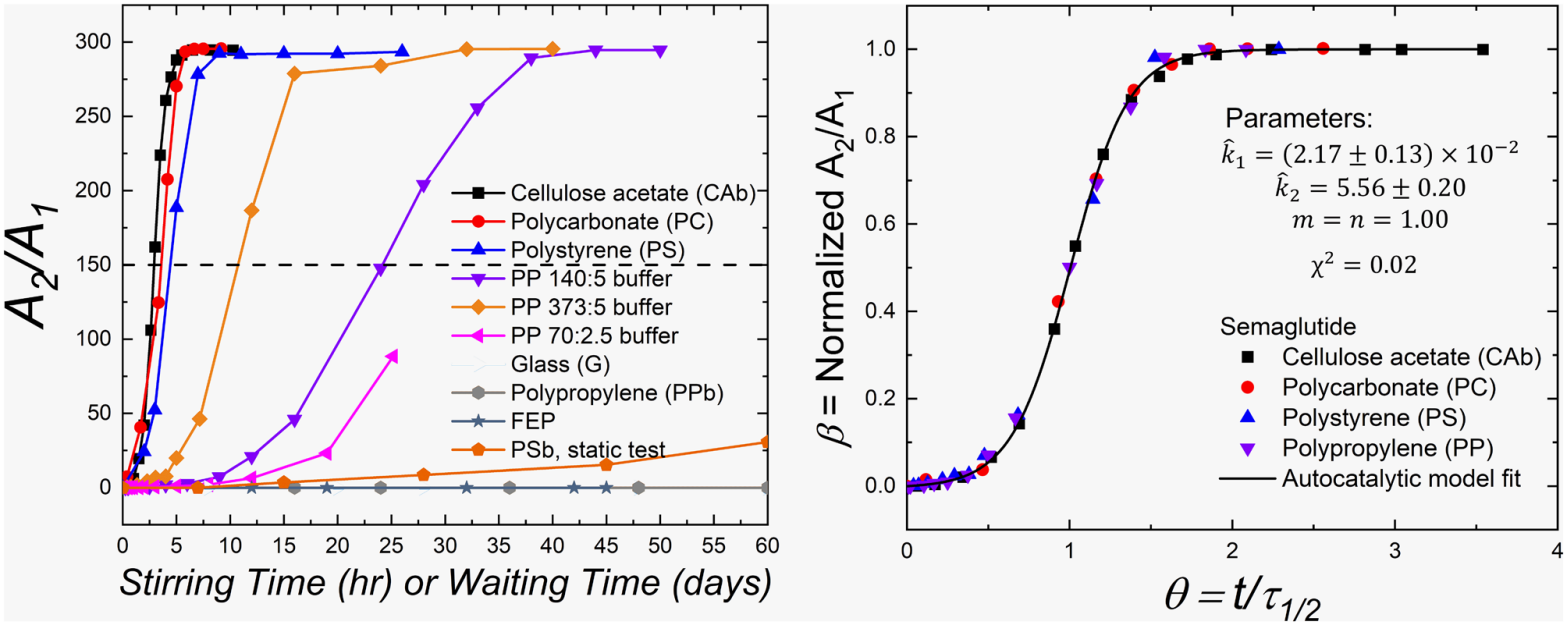
Microemulsion droplet formation rate: (*Left*) Relative area between large and small molecules from intensity-averaged size distribution (Fig. 2*B*) as a function of stirring time (for stirring tests) or waiting time (for static tests) for SMG in the presence of different surfaces, as well as varying salt conditions for PP: the ratios in the legend indicate the amount of sodium chloride: sodium phosphate in the buffer in mM. The connecting lines are for visual guide. The dashed line indicates the definition of $\tau_{1/2}$ . (*Right*) Nondimensionalized and normalized plot for SMG in regular buffer condition (140:5). The solid line is a fit to the autocatalytic reaction model (Eq. **5**) with the parameters and fitting quality.

The rates of ouzo formation were tracked by DLS using the areas identified for the oligomeric molecules and colloids in CONTIN intensity distributions, $A_{1}$ and $A_{2}$ , respectively (Fig. 4*B*). The variation of the ratio of integrated intensities $A_{2}/A_{1}$ , which is proportional to the intensity weighted relative amount of colloidal droplets formed ( $A_{2}$ ) compared with the amount of the oligomers ( $A_{1}$ ), as a function of stirring time for the various surface materials and buffer conditions are plotted in Fig. 6*A* (the autocorrelation functions can be found in *SI Appendix*, Fig. S5 along with the corresponding size distributions in *SI Appendix*, Fig. S6). Again, the ratio is not directly proportional to the mass rate of formation due to the complexities of the light scattering used to detect formation. However, as the size of the colloidal droplets is not dependent on surface or stirring rate, a direct comparison is possible. A sigmoidal shape is generally observed, but there is a very large difference in the formation rate across the experimental conditions. SMG in the presence of CAb exhibits the fastest emulsification rate, while the system in a PP container with low-salt buffer exhibited the slowest measurable rate. These differences in emulsification rate as well as the lack of ouzo formation in the G and FEP containers as well as for PPb agree with the aforementioned visual observations (Fig. 1).

The dynamic similarity of the kinetics of colloid formation suggests that the underlying physical processes are similar across all samples, but with varying rates. This dynamic similarity is demonstrated by a simple nondimensionalization of the experimental data. A characteristic half-time for formation, $\tau_{1/2}$   , is defined to be the time to reach the half of the maximum value of $A_{2}/A_{1}$   , indicated in the dash line in Fig. 6*A*. Replotting as the dimensionless ratio β   (normalized $A_{2}/A_{1}$   ) vs. dimensionless time $\theta =t/\tau_{1/2}$   , the results for samples CAb, PS, and PP at various buffer conditions are shown to follow a master curve in Fig. 6*B*, confirming dynamic similarity.

The sigmoidal-shaped increase in $A_{2}/A_{1}$   is typical of autocatalytic processes. The stable SMG oligomers form colloidal dispersions catalyzed by an appropriate interface. DLS measurements show that no size intermediate between the oligomers and colloids was observed, indicating the lack of slow growth mechanisms, such as Ostwald ripening. Hence, the colloidal droplets either form in solution very rapidly from nucleates that desorb from the surface, or form on the surface and detach into solution. In either case, it is reasonable to assume a two-step, surface-catalyzed process, as depicted in Fig. 3. An empirical, autocatalytic reaction model that is commonly used in the literature (32, 33) is used to fit this master curve and extract effective or overall reaction orders and kinetic constants (background for this model is found in *SI Appendix*):

[5] $$\frac{d\beta }{d\theta }=k_{1}{\left[(1-\beta )\right]}^{n}+k_{2}{\left[(1-\beta )\right]}^{n}{\left[\beta \right]}^{m},$$

where β(θ)   is our surrogate for time-dependent concentration, and $k_{1}$   and $k_{2}$   are dimensionless rate constants for the surface-mediated droplet formation and the droplet growth steps with reaction orders *n* and *m*, respectively. The model fit to the experimental master curve is found to be excellent, as shown in Fig. 6*B*. The fitting parameters m and n were found to be close to first-order reaction kinetics, which provides some confidence that a simple autocatalytic reaction sequence is a physically meaningful model for the process. Importantly, the observation that $k_{1}\ll k_{2}$ , indicates that the first step of surface-mediated droplet nucleation is the rate-limiting step. If this kinetic step is limited by desorption of a nucleation site for colloid formation, it would further explain why emulsification occurs much faster in stirring tests as compared with quiescent tests. Mechanical stirring may be reasonably expected to increase the rate of desorption of the nucleate or intermediate species from the surface, speeding up the whole process. Note that this modeling is not a rigorous study of the fundamental kinetic steps because the concentrations of nucleates, and colloidal droplets are only relative and not absolute. We model the overall kinetics using effective reaction orders and rate constants. However, the master curve and the autocatalytic reaction modeling provide evidence for the proposed two-step mechanism, establish a physical model for predicting the microemulsion rates for catalyzing surface materials, and may enable the prediction of ouzo formation from fitting initial, short-time measurements to the model for slowly evolving systems.

### Understanding the Role of the Specific Surface: Hansen Solubility Parameters (HSP).

The experimental observations and kinetic modeling confirm the critical role of surface-SMG interactions in controlling the instability. To better understand the role of this interaction, the wettability of the surfaces by SMG is quantitatively evaluated using the common framework of HSP (34, 35). Based on the Hildebrand solubility parameter approach, the total solubility (energy) is composed of three contributions: dispersion force, polar force, and hydrogen bonding.

[6] $$\delta^{2}=\delta_{D}^{2}+\delta_{P}^{2}+\delta_{H}^{2},$$

where δ is solubility parameter in ${\mathrm{MPa}}^{1/2}$ , related to cohesive energy ( E ) and molar volume ( V ) of the molecule ( $\delta^{2}=E/V$ ). The wettability between SMG and a surface material is evaluated by the relative energy difference (*RED*) between solubility parameter “distance” ( $R_{a}$ ) and the intrinsic solubility “range” of the surface material ( $R_{0}$):

[7] $$RED=\frac{R_{a}}{R_{0}}=\frac{1}{R_{0}}\sqrt{{4(\delta_{D,SMG}-\delta_{D,sur})}^{2}+{\left(\delta_{P,SMG}-\delta_{P,sur}\right)}^{2}+{\left(\delta_{H,SMG}-\delta_{H,sur}\right)}^{2}},$$

where $R_{0}$   is the radius of Hansen interaction sphere, which is a parameter specific to each surface material. Essentially, when the RED   is close to or less than unity, SMG can be expected to wet the surface. HSPs of SMG can be either determined experimentally (34) or calculated by a group contribution method as its chemical structure is known. Here, a group contribution method (31) developed specifically for pharmaceuticals was used for SMG and the results are presented in Table 2. The HSP and $R_{0}$   of the surface materials are taken directly from ref. 34 and are listed in Table 2, along with the calculated *RED*s. A few key points are summarized and discussed as follows:

**Table 2.** HSP (d-dispersion, p-polar, h-hydrophobic, and *R_0_*) for the materials of interest along with the calculated RED from Eq. **3**, where italic denote stable and bold denote unstable solutions

Materials	$\delta_{d}\;{MPa}^{1/2}$	$\delta_{p}\;{MPa}^{1/2}$	$\delta_{h}\;{MPa}^{1/2}$	$R_{0}$	RED	$1/\tau_{1/2}$ * h^−1^
SMG	17.7	2.2	15.1	–	–	–
*PTFE*	*16.2*	*1.8*	*3.4*	*3.0*	*3.1*	*0*
*FEP*	*19.0*	*4.0*	*3.0*	*4.0*	*3.1*	*0*
*PP (beads)*	*18.1*	*0.0*	*1.0*	*10.6*	*1.4*	*0*
**CA (beads)**	**17.1**	**13.1**	**9.4**	**10.6**	**1.2**	**0.34**
**PC**	**19.6**	**8.8**	**5.7**	**10.2**	**1.2**	**0.29**
**PS**	**21.3**	**5.8**	**4.3**	**12.7**	**1.1**	**0.22**
**PP container**	**20.3**	**6.3**	**5.4**	**10.6**	**1.1**	**0.04**

^*^$1/\tau_{1/2}$ values are from Fig. 6*A*.

1. Based on the observation of colloid formation in the presence of the different surface materials, an empirical criterion for the microemulsion effect in SMG is summarized as RED≤1.2 (Table 2). A RED value close to unity suggests SMG can wet the surface, exhibiting a SMG-philic behavior. When the RED value is larger, a SMG-phobic behavior can be expected, suppressing the instability. Hence, the SMG-philic surfaces promote ouzo formation while SMG-phobic surfaces suppress the instability. Such behavior agrees with previous reports of surface-catalyzed amyloid fibrillation (36), further supporting the two-step surface-mediated spontaneous emulsification as a valid hypothesis for this process.
2. The instability was observed when SMG is in the presence of a PP container but not when exposed to PP beads of similar surface area (Table 2). The difference is due to the existence of additives, e.g., inorganic fibers, used for manufacturing PP containers, which change the HSP of the surface material. The HSP of the fiber-filled PP is reported in ref. 34. The different observations between the PP beads and the PP container are consistent with the HSP analysis further supporting HSP analysis as a good candidate to predict the instability.
3. SMG was found to have a relatively small cohesive polar energy, but a high tendency for electron donation (hydrogen bonding). Note that the hydrogen-bonding component of the cohesive energy $\delta_{H}$ is being considered separately from the polar component. The high $\delta_{H}$ makes SMG water soluble, and the small value of $\delta_{P}$ indicates small intermolecular attractions (Keesom interactions).
4. The current HSP calculations are based only on chemical structure and do not explicitly consider any effects of buffer composition, which may affect the HSP. The presence of salts in the SMG solution screen electrostatic repulsion, making molecular attractions play a more important role in molecule interactions. Thus, the value of $\delta_{P,SMG}$ is anticipated to increase. The addition of sodium chloride and sodium phosphate is reported to weaken the hydrogen bonding strength (37), decreasing $\delta_{H,SMG}$ . Thus, the RED values are expected to decrease in buffer with added salts for all surface materials, which agrees with the observation that the kinetic rate of formation increases with added electrolytes. We remark that under the same buffer conditions, the addition of electrolytes is expected to affect the RED values to a similar degree across the different surface materials.
5. Furthermore, the rank order of the microemulsion formation rates or the rate of formation (shown in Table 2 in $1/\tau_{1/2}$   ) under stirring conditions, namely, CA>PC>PS>PP empirically follows the rank order of the difference in the polar component and not *RED*, which provides a hypothesis for further investigation and a basis for further molecular-level modeling.

The successful application of the HSP approach to correlate these data provides both a straightforward method for predicting the surface-mediated instability in formulations as well as a molecular thermodynamics framework for developing a better mechanistic understanding of the effect, both of which are of importance to various pharmaceutical processes. It may prove useful to further relate the surface-induced instability to some type of surface coacervation. Future studies will focus on surface adsorption behavior and peptide structure after desorption, which will help to establish a quantitative understanding of the phenomenon in acylated peptide solutions more broadly and help to distinguish the “ouzo-like” mechanism identified here from other types of surface-related instabilities.

In conclusion, a surface-mediated spontaneous emulsification was observed for the acylated peptide SMG in buffer solution, as summarized in Fig. 3. This type of physical instability is conceptually similar to the “ouzo” effect, but where colloid formation is driven by surface-related intermediate species rather than a non-solvent. The spherical shape, size, mass fractal internal structure, and the internal composition of the colloidal droplets were measured by combining light scattering and SAXS. From a Kratky plot and CD measurements, no loss of secondary structure in the peptide was detected for this physical instability. The size of the colloid formed was found to be independent of the surface material, but was affected by buffer conditions, i.e., salt concentration. The size and colloidal stability can be quantitatively predicted by the classical Rayleigh charge-limiting size model, revealing that the equilibrium colloidal size is controlled by the competition between surface tension and electrostatic forces. Although the rates of process differ for different surface materials, buffer conditions, and mechanical agitation, the process follows an empirical master curve that can be described by a standard autocatalytic reaction model. This dynamic similarity confirms that the emulsification kinetics follows a two-step mechanism where surface adsorption of SMG oligomers from solution is followed by subsequent desorption of a nucleate, with further, rapid growth of the intermediate species to form a colloidal dispersion. The mechanism is also supported semi-quantitatively by surface wettability analysis in the HSP framework. These experimental observations augmented by molecular thermodynamics and formation rate modeling provide a basis for possibly predicting similar ouzo-like formation in related formulations. Calculations suggest that the rate-limiting step of formation may be partly related to the polar component of the interaction between the active pharmaceutical ingredient and the surface, providing a basis for further research. This work provides a comprehensive understanding of and a prediction platform for this observation of surface-mediated spontaneous emulsification that is also valuable for assisting in reducing the API risk and drug product manufacturing failure.

## Materials and Methods

### SMG.

SMG consists of 31 amino acids in the main peptide chain whose sequence is available elsewhere (38). A C18 fatty di-acid side chain is conjugated to the peptide backbone. The molecular weight of an SMG monomer is 4,114 Da. SMG was purchased from PolyPeptide in lyophilized powder form. The powder as received was dispersed into phosphate buffer (sodium phosphate dibasic dihydrate, ${\text{Na}}_{2}{\text{HPO}}_{4}\cdot 2H_{2}O$ , 8 mM) with addition of sodium chloride (NaCl, 140 mM). Ultrapure water (Barnstead™ E-Pure™ Ultrapure Water Purification System, Thermo Scientific, Massachusetts, USA) was used, with a resistivity of 18.2 MΩ cm , total organic carbon below 10 ppb. The pH was adjusted to 7.4. The batch solution was made to have a SMG concentration of 20 mg/mL.

### Light Scattering.

SLS and multi-angle DLS experiments were performed using a LS spectrometer (LS Instrument®, Fribourg, Switzerland), equipped with a diode laser light source of λ=660 nm   . The modulated 3D Cross-Correlation function (39) was used for turbid samples. For DLS experiments, 5-mm high-throughput NMR tubes are used as sample cells (Wilmad-LabGlass, New Jersey, USA). DLS experiments were taken at a scattering angle of 90° to minimize the scattering contributions from any dust as well as rotational diffusion. Before each measurement, the self-diffusion limit is confirmed by matching the initial values of the intensity autocorrelation function, $g_{2}\left(t\right)-1$   , between the 2D and 3D modes of the instrument. An averaging time of 8 min was chosen to ensure a good signal-to-noise ratio. DLS measures the fluctuation of the scattering intensity, characterizing it by $g_{2}\left(t\right)-1$   , which is directly related to the diffusion coefficient. Then, the hydrodynamic sizes are calculated from the Stokes–Einstein–Sutherland equation (18, 19). CONTIN analysis was performed to yield the intensity-averaged size distributions, $F(d_{H})$ . SLS experiments use standard borosilicate 10 × 75-mm glass culture tubes (Thermo Fisher Scientific Inc., Massachusetts, USA). SLS experiments were performed over a range of scattering angles from 10° to 150°. The refractive index measurements were performed using a Abbemat 300 refractometer (Anton Paar Inc., Virginia, USA), with a wavelength of 589 nm. For the SLS in the Guinier regime ( $qR_{g}<1.8$ ), $R_{g}$ can be determined from a linear fitting of lnI(q) vs. $q^{2}$:

[8] $$\text{ln}I\left(q\right)=\text{ln}I_{0}-\frac{q^{2}R_{g}^{2}}{3}.$$

### Zeta Potential.

The zeta potential is measured on a Malvern Zetasizer Pro (Malvern Panalytical Inc., Massachusetts, USA), with folded capillary zeta cell (DTS1070).

### SAXS.

SAXS was performed at BioCAT (beamline 18ID at the Advanced Photon Source, Chicago, with a wavelength of $\lambda_{\mathrm{SAXS}}=1.03\;Å$ ). Samples were flown through the SAXS flow cell, which consists of a 1.0-mm ID quartz capillary with ~20 μm walls. A coflowing buffer sheath is used to separate the sample from the capillary walls, helping prevent radiation damage (40). X-ray energy for the measurements was 12 keV. Scattering intensity was monitored using an Eiger2 XE 9M (Dectris) detector which was placed 3.6 m from the sample, allowing a q-range of 0.003 to 0.42 ${Å}^{-1}$ . 0.5 s exposure time was used with a 1 s total exposure period and data was reduced using BioXTAS RAW 2.1.1 (41).

### CD.

CD measurements were performed at 25 °C on a Jasco J-1500 (Jasco Inc., Tokyo, Japan) with a wavelength range from 190 to 250 nm. A 1-mm path length quartz cuvette (Hellma, Müllheim, Germany) was used at a scanning speed of 50 nm/min and a bandwidth of 1 nm. Samples were diluted to 0.2 mg/mL for signal accuracy required by the instrument. Each CD curve is an average of three measurements. The baseline (buffer solution) was subtracted from all spectra.

### Stirring Test and Static Test.

SMG emulsification under static and stirring conditions was performed in the presence of varying surfaces. For both tests, a SMG solution was placed into a testing container of specific material of interest with a stirring bar [polytetrafluoroethylene (PTFE) surface, 20 mm in length], or a glass container (7 mL SMG) containing polymer beads of a specific material of interest (118 PSb or 140 CAb) together with a stirring bar. For the stirring test, samples were stirred at 250 rpm and 25 °C. For the static test, samples were held quiescently in a container at 25 °C. Aliquots of sample (~250 μL) were removed for DLS testing at selected time. Before each DLS test, the sample was allowed to stabilize for 30 min. For the tests in different containers or with different polymer beads embedded, the surface-to-solution volume ratio (StV) was set to be 1.0 cm^−1^. Six different materials of interest were tested, and their information is listed below, together with the amount of SMG in volume used in the container to keep a same StV:

1. Glass container (G) (20-mL WHEATON® liquid scintillation vial. Product number 986546, Wheaton Industries, Inc., New Jersey, USA).
2. Cellulose acetate (CAb) beads (white 1.41-mm spheres, item # CAS-ALA-1.3 1.41 ± 0.05 mm-100, Cospheric LLC, California, USA).
3. PC container (70-mL centrifuge bottle, product number 355655, Beckman Instruments Inc., California, USA), using 50 mL SMG solution.
4. PS container (250-mL storage bottle, product number 430281, Corning, New York, USA), using 82 mL SMG solution; PSb (1.55-mm spheres, item # PSS-1.05 1.55 ± 0.05 mm-100, Cospheric LLC, California, USA).
5. Polypropylene (PP) container (50-mL Basix centrifuge tube, catalogue number 14-955-239, Thermo Fisher Scientific Inc., Massachusetts, USA), using 21 mL SMG solution; Polypropylene beads (PPb) (product # 428175, Sigma-Aldrich, Missouri, USA).
6. FEP container (3 mL international space station sample bag, Techshot Inc., Indiana, USA).

## Supplementary Material

Appendix 01 (PDF)

## Data Availability

There are no data underlying this work.
