# Supplementary material for: Surface-mediated spontaneous emulsification of the acylated peptide, semaglutide
Source: Proc Natl Acad Sci U S A. 2024 Jan 16;121(5):e2305770121. doi: 10.1073/pnas.2305770121 (PMC10835113; doi:10.1073/pnas.2305770121)
Supplement: Supplementary file 1 — Appendix 01 (PDF) [file pnas.2305770121.sapp.pdf]

Supporting Information for

Surface-mediated spontaneous emulsification of the acylated peptide, semaglutide

Qi Li[1], Vasudev Tangry[1], David P. Allen[2], Kevin D. Seibert[2], Ken K. Qian[2,*], Norman J. Wagner[1,*]

[1] Center for Neutron Science, Department of Chemical and Biomolecular Engineering Department, University of Delaware, Newark, DE 19716

[2] Eli Lilly and Company, Indianapolis, IN 46225

Email: qian_ken_k@lilly.com, wagnernj@udel.edu

This PDF file includes:

Supporting text
Figures S1 to S6
SI References

Supporting Information

Glossary of definitions

Self-association, secondary structure: Protein or peptide "adopt a local folding referred to as the secondary structure (i.e., α-helix or β-sheet structure) and a tridimensional location of the secondary structure in space, known as the tertiary structure. Moreover, some proteins adopt a quaternary structure, which implicates the association between protein subunits and their arrangements from dimers to oligomers[1]. The self-association of proteins in different oligomeric states is mainly governed by nonbonded interactions such as the van der Waals forces, hydrogen and ionic bonds and π–π interactions[2]. However, in some cases, the formation of specific disulfide bonds is critical for protein oligomerization[1]."

Oligomer molecule[3]: refers to a molecular complex of intermediate molar mass that is comprised of a small plurality of repeated molecular units that are bonded either covalently or physically.

Microemulsion[4]: "Microemulsions are dispersions of two or more immiscible or partially miscible fluids stabilized by added surfactants. The dispersed domains are generally in the nanometer size range. Visually, they are transparent or translucent, and their appearance does not change with time. Microemulsions are considered to be thermodynamically stable."

Spontaneous emulsification[5]: "Generally, spontaneous emulsification occurs when two immiscible liquids emulsify without the aid of any external thermal or mechanical energy source. Depending on the nature of the liquids involved, it may take from a few minutes to several days for the process to complete."

Ouzo effect[6]*:* refers to the spontaneous emulsification leading to a stable, milky colloidal solution that derives its name from the Greek beverage.

Visual observations in stirring test

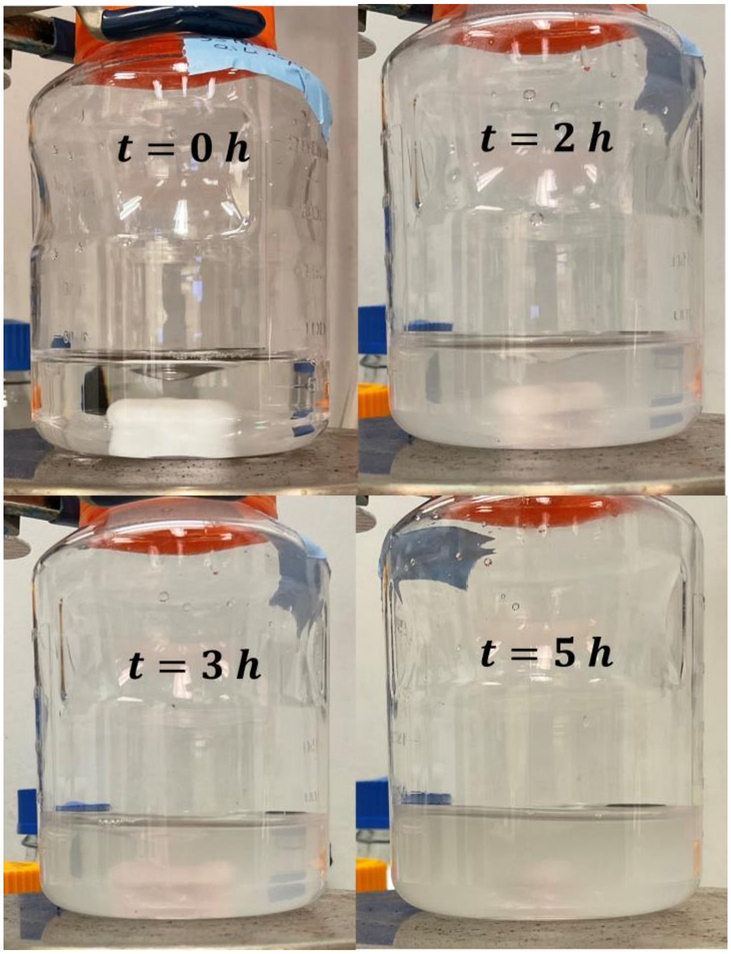

Fig. S1. Pictures of 20 *mg/mL* semaglutide (SMG) in buffer stirring in a polystyrene (PS) container with a polytetrafluoroethylene bar embedded at 25 *°C*. Stirring rate is 250 *rpm*.

The SMG solution in the PS container became cloudy with time under stirring. From $t = 0\ h$ to $t = 5\ h$, the turbidity of the sample increases. After 5 hours, no visual difference was observed but light scattering experiments show that the number of colloidal droplets continually increases.

Grahame equation.

The relationship between the surface charge density (σ) and surface potential, which is normally taken as zeta potential (ψ_0) is determined from the electrolyte concentrations via the Grahame equation[7]:

$$\sigma = \sqrt{8\varepsilon_0 \varepsilon kT} \sinh\{e\psi_0/(2kT)\}\left\{[Cl^-]_\infty + [HPO_4^{2-}]_\infty\left(2 + e^{\psi_0/(kT)}\right)\right\}^{1/2} \quad \text{(S1)}$$

where $\varepsilon_0 = 8.85 \times 10^{-12}\ F \cdot m^{-1}$ is the vacuum permittivity. $\varepsilon = 78.3$ is dielectric constant (relative permittivity) of the buffer. $e = 1.6022 \times 10^{-19}\ C$ is the elementary charge.

Kratky plot of SMG from SAXS.

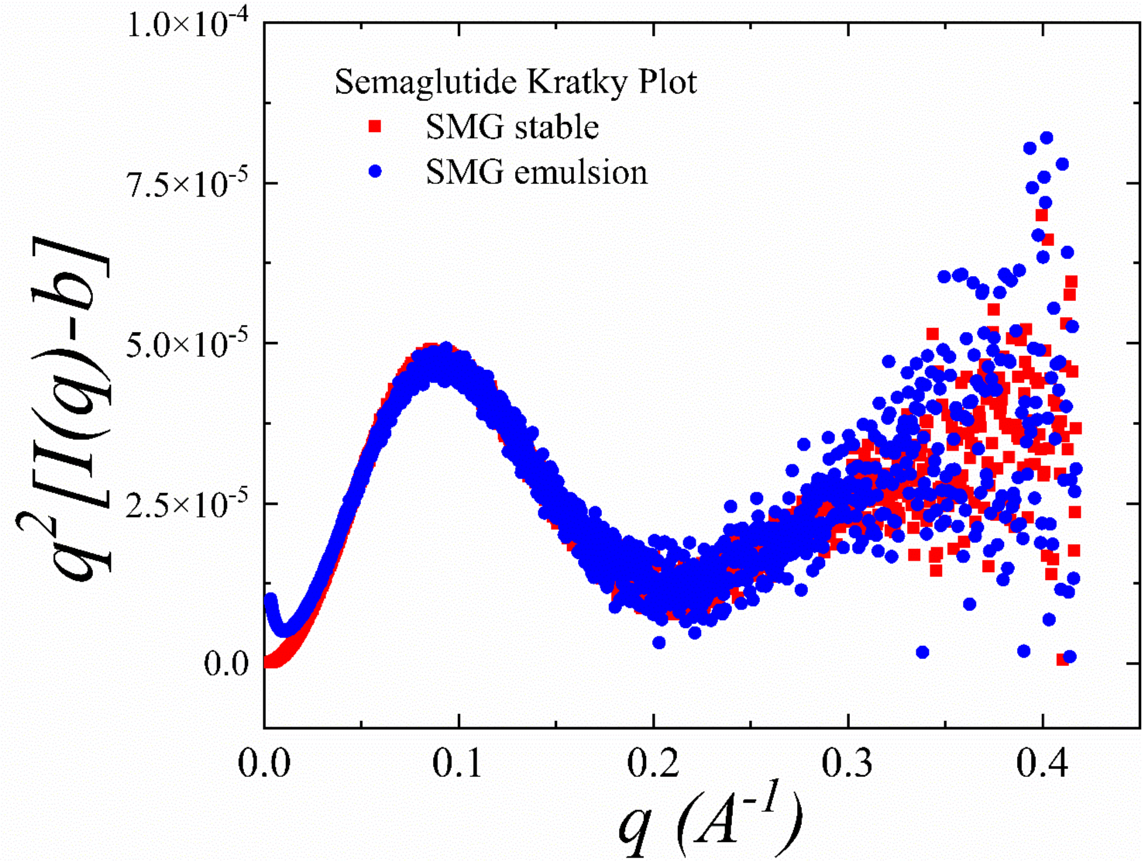

Fig. S2. Kratky plot ($q^2[I(q) - b]$ $vs. q$) of the SAXS data of SMG stable solution (red squares) and SMG emulsion (blue circles). Both the stable solution and emulsion are at 2 *mg/mL*. The emulsion is from 20 *mg/mL* SMG stirring test in PS container for 16 hours at 25 ^oC and then diluted to 2 *mg/mL*.

The Kratky plot is commonly used to assess the degree of unfolding in peptide or protein solutions[8]. By plotting $q^2I(q)$ versus q, folded peptide or protein molecules exhibit a Gaussian peak but no high-q plateau. Unfolded peptides should show a plateau at high-q, and the coexistence of the Gaussian peak and high-q plateau can be used to estimate the amount of unfolding. In Figure S2, for both the stable and emulsion samples, Gaussian peaks are observed around $q = 0.09\ A^{-1}$, with no high-q plateau evident, indicating a folded peptide structure in both samples. Note that the large emulsion droplets are evident as the upturn in the plot at very small q on this linear scale, which has no effect on the analysis of the dispersed peptides.

Polydisperse sphere model fitting for SAXS

The polydisperse sphere model describes the scattering intensity via form factor, $P(q)$, following the formulation by Guinier[9] (full description can be found in SasView documentations[10]):

$$P(q) = \frac{scale}{V_s} F^2(q) + background \qquad \text{(S2)}$$

and

$$F(q) = \frac{3}{V_s}\left[(\rho_s - \rho_{sol})\frac{\sin(qr_s) - qr_s\cos(qr_s)}{(qr_s)^3}\right] \qquad (S3)$$

where V_s is the volume of the sphere and $r_s$is the radius of the sphere is the SLD of the shell, respectively; ρ_{sol} is the SLD of the solvent. SLD measures the scattering capability of a material, therefore is related to the chemical structure and density of the material:

$$SLD = \frac{\rho N_a \sum_{i=1}^{N} b_i}{\sum_{i=1}^{N} M_i} \qquad (S4)$$

where ρ is the density of the material. N_a is the Avogadro number. The sum of b_i and M_i correspond to the scattering length contributions and molecular weight contributions of all the chemical elements in the material, respectively. For the current work, the SLDs are calculated from SasView: $SLD_{water} = 9.40 \times 10^{-6}Å^{-2}$, $SLD_{buffer} = 9.45 \times 10^{-6}Å^{-2}$ and $SLD_{SMG} = 1.06 \times 10^{-5}Å^{-2}$ with $\lambda_{SAXS} = 1.03$ Å, $\rho_{water} = 1.00\ g/cm^3$, $\rho_{NaCl} = 2.16\ g/cm^3$, $\rho_{sodium\ phosphate} = 2.54\ g/cm^3$. The density of SMG was measured by density meter (DDM 2911, Rudolph Research Analytical, New Jersey, USA) to be $\rho_{SMG} = 1.16 \pm 0.04\ g/cm^3$.

The polydisperse model fit is shown in Figure 5a with fitting parameters of $scale = 2.4 \times 10^{-3}$, $background = 2.20 \times 10^{-4}\ cm^{-1}$, $r_s = 20.6$ Å, $\rho_s = 10.6 \times 10^{-6}Å^{-2}$, $\rho_{sol} = 9.48 \times 10^{-6}Å^{-2}$, with a polydispersity ratio of 0.16 (lognormal) and the fitting error to be $\chi^2 = 4.8 \times 10^{-7}$. We note that the SMG concentration is 2 mg/ml so the scale factor is within 20% of expectation. Further, for a uniform, solid sphere, the radius of gyration is $\sqrt{\frac{3}{5}} * r_s \approx 16$ Å , which is in agreement with the radius of gyration determined from SLS (16.6±4.5 Å) given the polydispersity.

Zimm plot of stable SMG solution

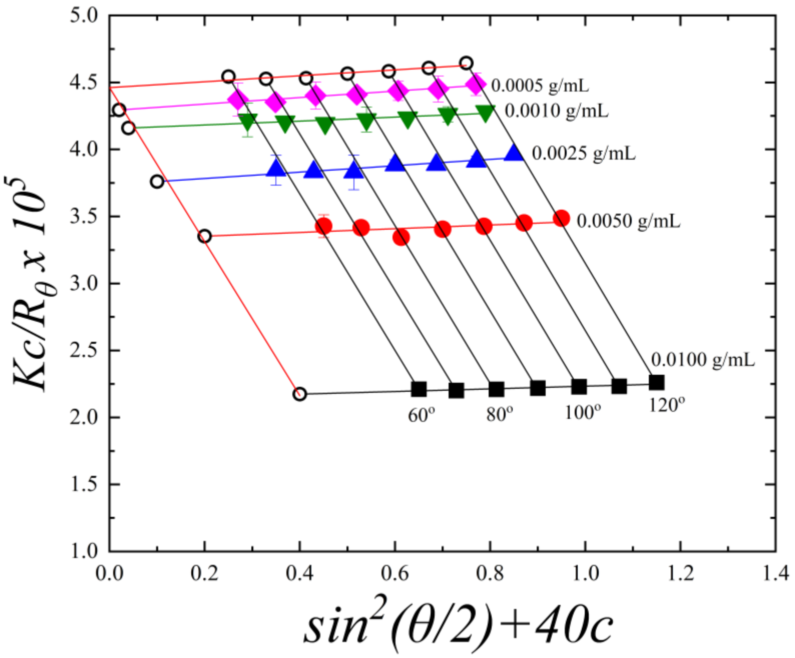

Fig. S3. Zimm plot of SMG measured by static light scattering. Different concentrations are from diluting the 20 mg/mL SMG with standard buffer. SLS was measured from 60° to 120° at an interval of 10°.

In the Guinier region, by measuring the scattering intensity of different concentrations at varying scattering angles, the zero-concentration and zero-angle scattering information can be extracted, which contains information of molecular weight (M_w), radius of gyration (R_g) and second Virial coefficient (A_2). A typical Zimm plot analysis follows the following relationship

$$\frac{Kc}{R\theta} = \frac{1}{M_w}\left(1 + \frac{R_g^2}{3}q^2\right)(1 + 2A_2c) \tag{S5}$$

where $K = \frac{4\pi^2 n^2 \left(\frac{dn}{dc}\right)^2}{N_0 \lambda^4}$ is an optical constant, n denotes refractive index (SMG oligomer at 20 mg/ml is 1.342 ±0.003), c is the sample concentration, λ is the laser wavelength, and R_θ is the excess Rayleigh ratio.

Figure S3 presents the Zimm plot analysis on a stable SMG solution. Due to the small size of the stable SMG molecules, limited angular dependence is observed. The crossover point of the red solid lines for the two sets of extrapolated data (empty spheres) gives a molecular weight of $(2.24 \pm 0.46) \times 10^4 g \cdot mol^{-1}$, suggesting a tetramer to hexamer self-association oligomer structure of the stable SMG molecule. The slope of the zero-concentration line entails the radius of gyration of the molecule, $R_g = 1.66 \pm 0.45\, nm$, which agrees with the Guinier analysis result from SAXS measurement ($R_g = 1.87 \pm 0.22\, nm$).

Zimm plot of SMG colloidal solutions

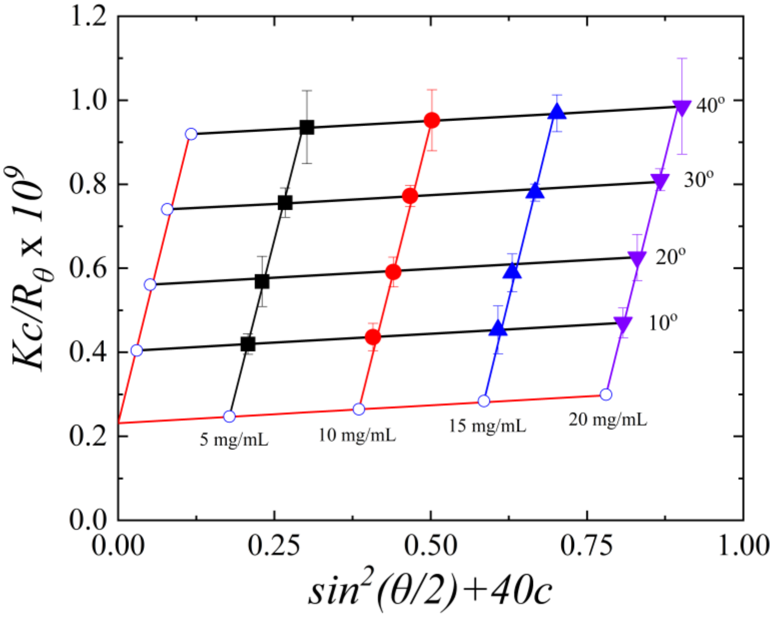

Fig. S4. Zimm plot of SMG colloids measured by static light scattering. Different concentrations are from diluting the 20 mg/mL SMG with standard buffer. SLS was measured from 10° to 40° at an interval of 10°.

Zimm plot analysis on the SMG colloids (Figure S4) reveals the internal composition of the emulsion droplets. The refractive index of the SMG colloids at 20 mg/ml is 1.344 ±0.002), The intercept on the y-axis gives $\frac{1}{M_w}$, so we have $M_w = (4.3 \pm 1.8) \times 10^6\ kDa$. The R_g from Zimm plot shows $212.8 \pm 30.1\ nm$, agreeing with that from Guinier analysis in SLS experiments. The slope of the zero-q line gives the second Virial coefficient $(A_2 \approx 6A_{2,hard\ sphere})$ where $A_{2,hard\ sphere} = \frac{2\pi d^3}{3}$ with d being the particle diameter[11], taking the value of the hydrodynamic diameter of the microemulsion droplet in standard buffer ($d_H = 514.5\ nm$).

Intensity autocorrelation function

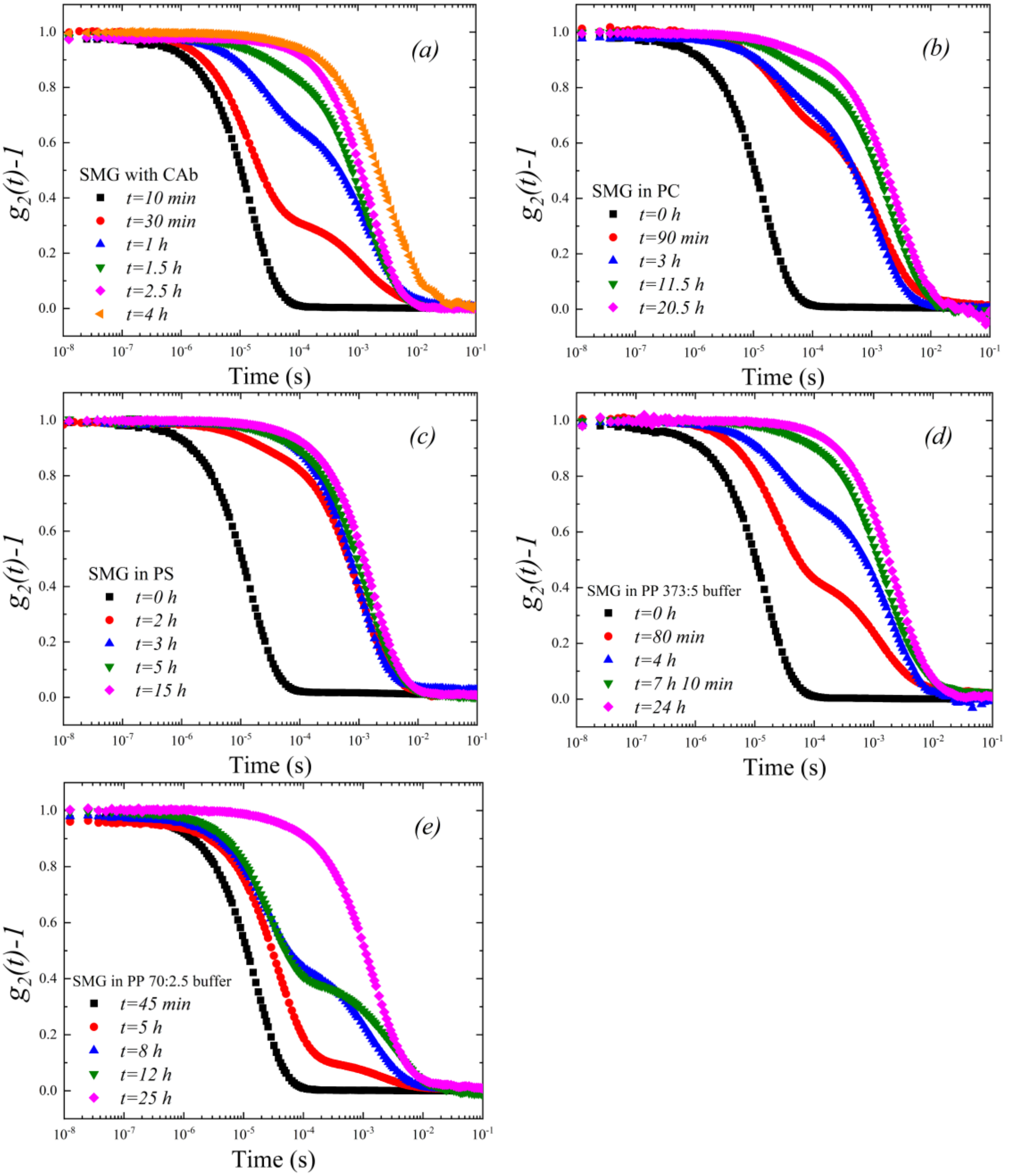

Fig. S5. Intensity autocorrelation function, $g_2(t) - 1$, as a function of time from dynamic light scattering measurements (LS Spectrometer) for 20 mg/mL SMG stirring tests in the presence of different surface materials or different buffer conditions. Different curves in a same plot represent different stirring time. The stirring tests were done at 25 ^{o}C and the stirring rate is 250 rpm. **(a)** cellulose acetate beads (CAb); **(b)** polycarbonate (PC); **(c)** polystyrene (PS); **(d)** polypropylene in 373 mM NaCl and 5 mM sodium phosphate buffer; **(e)** polypropylene in 70 mM NaCl and 2.5 mM sodium phosphate buffer.

The intensity autocorrelation function, $g_2(t) - 1$, is calculated by instrument software from the fluctuation in scattering intensity:

$$g_2(t) = \frac{\langle I(0)I(t)\rangle}{\langle I(0)\rangle^2} \quad \text{(S6)}$$

The ACFs shown in Fig S5(a)-(e) indicate that with increasing stirring time, a population of larger objects appears, as indicated by the developing shoulder in the long-time regime. With the information of $g_2(t) - 1$ and the Stokes-Einstein-Sutherland equation $D_o = \frac{k_b T}{6\pi\mu_o a}$* relating the diffusion coefficient to the particle size a and suspending medium viscosity μ_o, CONTIN analysis can be carried out to obtain the size distribution (Fig. S6).

---

* We note that the Australian Physicist, William Sutherland, independently and concurrently developed the commonly termed Stokes-Einstein equation and so it is more properly termed the Stokes-Einstein-Sutherland equation. More about the fascinating history of this seminal contribution to science can be found on various historicial online sites, including : http://www.ph.unimelb.edu.au/~dnj/wyop/wyop2005-sutherland-essay.html

DLS size distributions

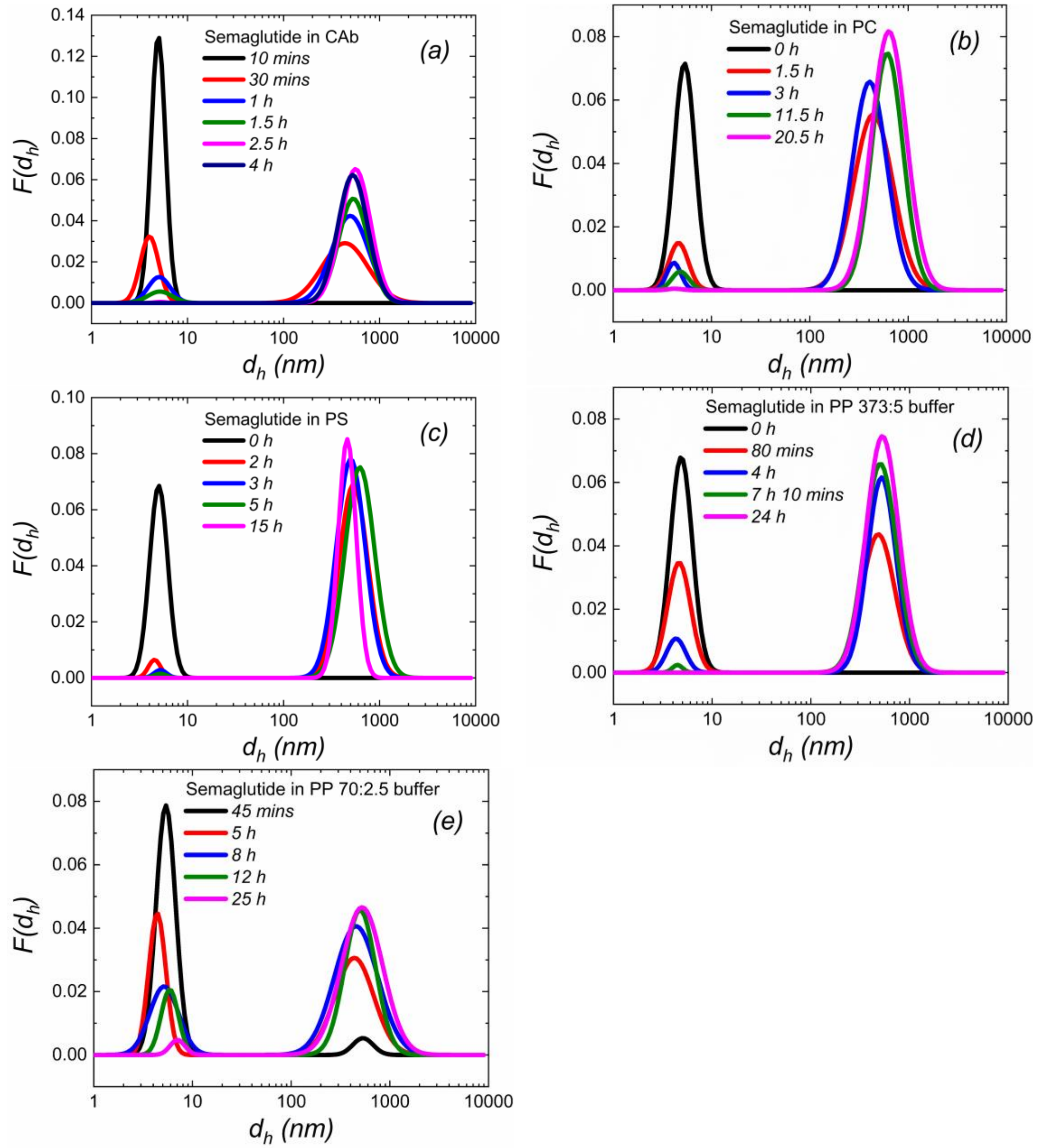

Fig. S6. Intensity-averaged size distributions of 20 *mg/mL* SMG stirring tests in the presence of difference surfaces, calculated from Fig. S5 using CONTIN algorithm. The surface materials from (a) to (e) correspond to the same order as in Fig. S5.

Autocatalytic reaction model

α

The autocatalytic reaction model is commonly used in modeling processes assume having a two-step mechanism: autocatalytic nucleating and nucleate growth. Examples of its application include polymerization[12,13], amyloid aggregation[14,15], and metal clustering[16]. The model can be described by:

$$A \xrightarrow{k_1} B$$
$$A + B \xrightarrow{k_2} B$$
$$\frac{d[B]}{dt} = k_1[A]^n + k_2[A]^n[B]^m$$

where the first chemical reaction denotes the process of monomer or stable molecule becoming nuclei and the second reaction describes the growth of the nucleus. The equation shows the reaction kinetics form of the model. The non-dimensional form of the equation is shown in the main text as Eqn (5).

S.I. References

1. S. Liu, A review on protein oligomerization process. *International Journal of Precision Engineering and Manufacturing* **16**, 2731-2760 (2015).
2. A. Dhotel *et al.*, Molecular motions in functional self-assembled nanostructures. *Int J Mol Sci* **14**, 2303-2333 (2013).
3. A. D. McNaught, A. Wilkinson, Eds., *IUPAC. Compendium of Chemical Terminology (the "Gold Book").* (Blackwell Scientific Publications, Oxford, 1997), 2nd Ed.
4. J. Eastoe, M. H. Hatzopoulos, R. Tabor, "Microemulsions" in Encyclopedia of Colloid and Interface Science, T. Tadros, Ed. (Springer Berlin Heidelberg, Berlin, Heidelberg, 2013), 10.1007/978-3-642-20665-8_25, pp. 688-729.
5. J. C. López-Montilla, P. E. Herrera-Morales, S. Pandey, D. O. Shah, Spontaneous Emulsification: Mechanisms, Physicochemical Aspects, Modeling, and Applications. *Journal of Dispersion Science and Technology* **23**, 219-268 (2002).
6. S. A. Vitale, J. Katz, Liquid Droplet Dispersions Formed by Homogeneous Liquid−Liquid Nucleation: “The Ouzo Effect”. *Langmuir* **19**, 4105-4110 (2003).
7. J. N. Israelachvili, *Intermolecular and Surface Forces* (Elsevier Science, 2011).
8. V. M. Burger, D. J. Arenas, C. M. Stultz, A Structure-free Method for Quantifying Conformational Flexibility in proteins. *Sci Rep* **6**, 29040 (2016).
9. A. Guinier, G. Fournet, *Small-Angle Scattering of X-Rays* (John Wiley & Sons, New York, 1955).
10. M. Doucet *et al.* (2017) SasView version 4.1.2. (https://www.sasview.org/).
11. H. Hadwiger, Altes und Neues über konvexe Körper. *The Mathematical Gazette* **40**, 310 (1955).
12. S. A. Madbouly, J. U. Otaigbe, Kinetic Analysis of Fractal Gel Formation In Waterborne Polyurethane Dispersions Undergoing High Deformation Flows. *Macromolecules* **39**, 4144-4151 (2006).
13. B. Lucio, J. L. de la Fuente, Kinetic and thermodynamic analysis of the polymerization of polyurethanes by a rheological method. *Thermochimica Acta* **625**, 28-35 (2016).
14. R. Sabaté, M. Gallardo, J. Estelrich, An autocatalytic reaction as a model for the kinetics of the aggregation of beta-amyloid. *Biopolymers* **71**, 190-195 (2003).
15. I. A. Iashchishyn, D. Sulskis, M. Nguyen Ngoc, V. Smirnovas, L. A. Morozova-Roche, Finke-Watzky Two-Step Nucleation-Autocatalysis Model of S100A9 Amyloid Formation: Protein Misfolding as "Nucleation" Event. *ACS Chem Neurosci* **8**, 2152-2158 (2017).
16. M. A. Watzky, R. G. Finke, Transition Metal Nanocluster Formation Kinetic and Mechanistic Studies. A New Mechanism When Hydrogen Is the Reductant: Slow, Continuous Nucleation and Fast Autocatalytic Surface Growth. *Journal of the American Chemical Society* **119**, 10382-10400 (1997).
